# Littoral Slope, Water Depth and Alternative Response Strategies to Light Attenuation Shape the Distribution of Submerged Macrophytes in a Mesotrophic Lake

**DOI:** 10.3389/fpls.2019.00169

**Published:** 2019-02-20

**Authors:** Liang He, Tianshun Zhu, Yao Wu, Wei Li, Huan Zhang, Xiaolin Zhang, Te Cao, Leyi Ni, Sabine Hilt

**Affiliations:** ^1^Donghu Experimental Station of Lake Ecosystems, Institute of Hydrobiology, Chinese Academy of Sciences, Wuhan, China; ^2^Key Laboratory of Poyang Lake Environment and Resource Utilization, Ministry of Education, Nanchang University, Nanchang, China; ^3^Institute of Ecology and Environmental Science, Nanchang Institute of Technology, Nanchang, China; ^4^Leibniz-Institute of Freshwater Ecology and Inland Fisheries, Berlin, Germany

**Keywords:** submerged macrophyte, littoral slope, water depth, response strategies, species distribution

## Abstract

Light is a major limiting resource in aquatic ecosystems and numerous studies have investigated the response of submerged macrophytes to low light conditions. However, few studies have tested whether different light response strategies can also have consequences for macrophyte distribution along different littoral slopes in lakes, which are known to affect macrophyte biomass due to differences in drag forces and sediment characteristic. In this study, we tested (1) whether two macrophyte species of different growth forms (canopy-forming: *Potamogeton maackianus*, rosette-type: *Vallisneria natans*) differ in their response strategies to low light conditions and (2) how these responses influence their distribution along different basin slopes in the mesotrophic Lake Erhai, China. We hypothesized that the canopy-forming species responds to low light conditions at deeper sites by stem elongation while the rosette-type species increases its shoot chlorophyll content. As a consequence, *P. maackianus* should have a higher susceptibility to drag forces and thus prevail at sites with lower slopes. Sites with higher slopes should offer a niche for rosette-type species like *V. natans* that can better withstand drag forces. We surveyed the distribution and abundance of the two macrophyte species at 527 sampling points along 97 transects in Lake Erhai and measured their height, leaf and stem/rhizome biomass, and leaf chlorophyll *a* content at different water depths. Our results confirmed stem elongation as a strategy to low light conditions by the canopy-forming species *P. maackianus*, while *V. natans* produced more chlorophyll *a* per shoot biomass at deeper sites to tolerate shading. As hypothesized, these alternative response strategies to low light conditions resulted in a trade-off regarding the plants ability to grow at different basin slopes. *P. maackianus* was dominant at sites with low-moderate slope (0–4%) and low-moderate water depth (2–4 m), while sites with high basin slope (4–7%) combined with moderate-high water depth (3–5 m) were dominantly colonized by *V. natans*. The latter habitat thus represents a potential refuge for rosette-type macrophyte species that are often outcompeted when shading increases during eutrophication.

## Introduction

Light limitation has driven the evolution of highly plastic adaptive strategies in plants to either tolerance or avoidance of shading by neighboring vegetation (Franklin, 2008). In freshwater ecosystems, roughly 10% of the global radiation is reflected at the air-water interface and a significant portion is attenuated with depth resulting in low light conditions in most deep aquatic habitats (Spence, 1981). Consequently, numerous aquatic plants have evolved a high plasticity in morphological and physiological traits in response to low light conditions (Maberly, 1993; Olesen et al., 2002). Previous studies showed that the response strategy of submerged macrophytes to low light conditions largely depends on their growth form (Chambers and Kalff, 1987; Fu et al., 2012). Canopy-forming species, such as *Myriophyllum spicatum* and *Potamogeton wrightii*, tend to allocate more biomass to stems, grow taller and form dense canopies to counter light attenuation in the water column (Strand and Weisner, 2001; Fu et al., 2012). In contrast, rosette-type macrophytes such as *Vallisneria* species can increase their plant height in deeper sites as well (Fu et al., 2012), but mainly tolerate shading through a lower light compensation point and a higher leaf mass ratio of total plant mass compared to canopy-forming macrophytes (Su et al., 2004; Chen et al., 2016).

Increasing eutrophication of lakes leads to increased shading of submerged macrophytes by phytoplankton and periphyton. As a consequence, small species such as charophytes and rosette-type angiosperms disappear and tall, canopy-forming macrophytes that can escape low light conditions become dominant (Sand-Jensen et al., 2008; He et al., 2015; Hilt et al., 2018). However, other factors such as morphometric characteristics of the littoral area additionally may influence the biomass and community structure of submerged macrophytes in lakes (Kolada, 2014). Littoral slope for example was suggested as a good predictor of the maximum biomass of submerged macrophyte communities (Duarte and Kalff, 1986). Low slope areas usually have fine, nutrient-rich sediments and little water movement, while coarse, nutrient-poor sediments and stronger water movement characterize high slope areas in temperate lakes (Håkanson, 1977; Duarte and Kalff, 1986). In aquatic environments, hydrodynamic forces caused by water movement can be many times the drag forces produced by wind on land (Puijalon et al., 2011). Currents and waves can cause strong damage to or even uproot submerged plants (Bornette and Puijalon, 2011). However, the degree of damage depends on the sediment characteristic (Barko and Smart, 1986; Schutten et al., 2005; Spierenburg et al., 2013) and the macrophyte’s ability to resist breakage and uprooting, which is closely related to its size and shape (Schutten and Davy, 2000; Schutten et al., 2004). Generally, short rosettes with linear soft leaves are better at resisting drag forces compared to tall canopy-forming submerged macrophytes at a given water velocity (Puijalon et al., 2011). Hence, short rosette-type species may be better adapted to disturbance by water movement. In addition, canopy-forming submerged macrophytes need more nutrients to support their high growth rates, while short rosette-type species grow slowly and can survive under lower nutrient supply (Chambers, 1987). Therefore, the canopy-forming species may dominate in low slope areas of mesotrophic lakes, while rosette-type species can better tolerate the stress of higher slopes.

Although numerous studies have investigated the response of macrophytes to low light conditions, only a few of them have tested whether these response strategies to shading conditions affect the distribution of macrophytes along other environmental gradients such as sediment characteristic and drag forces (Spierenburg et al., 2013). In this study, we measured different morphological and physiological parameters and the abundance of two dominant macrophyte species, the canopy-forming *P. maackianus* and the rosette-type species *V. natans* at different water depths (determining light availability) and littoral slopes (determining sediment characteristic and drag forces) in the mesotrophic Lake Erhai, China. We tested (1) whether the two species show the response strategies to low light conditions typical for canopy- and rosette-type macrophytes and (2) whether these responses influence their distribution along different basin slopes. We hypothesized that the canopy-forming species *P. maackianus* responds to low light conditions in deeper water by stem elongation while *V. natans* increases its shoot chlorophyll content. As a consequence, *P. maackianus* should have a higher susceptibility to drag forces and thus prevail at sites with lower slopes. In contrast, the rosette-type species *V. natans* should tolerate higher drag forces and thus be prevalent at sites with higher slopes.

## Materials and Methods

### Study Location and Macrophyte Species

**FIGURE 1:**
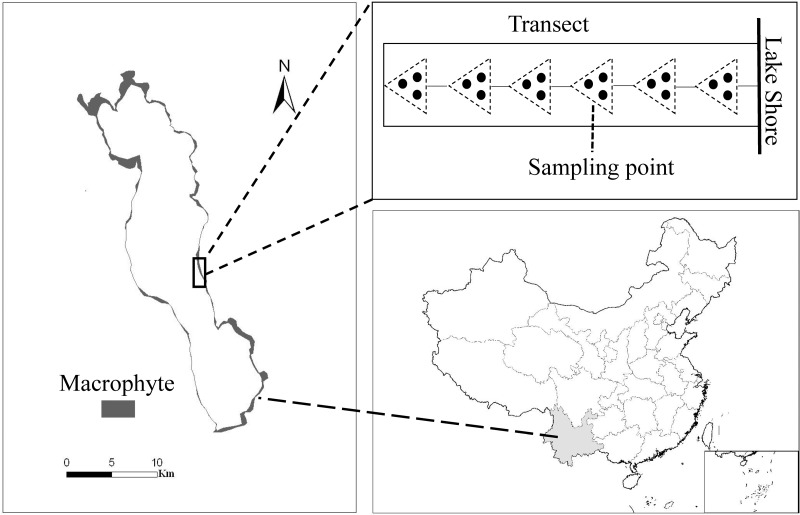
Macrophyte coverage in Lake Erhai (left), location of the lake in China (below) and sampling scheme at each transect (above). The small rectangle at the bottom right shows the South China Sea Islands.

The study was carried out in the mesotrophic Lake Erhai (25°52′N, 100°06′E), located in the Yunnan province of China (Figure 1). The lake has a total area of 249 km^2^, a moderate water depth (maximum depth 20.5 m, mean depth 10.5 m) and a large variation in littoral slopes. Macrophytes show a zonation along the water depth gradient, with most of the 12 submerged species inhabiting shallow water (0–3.0 m depth), and only a few species extending to deeper water (Fu et al., 2014). Maximum colonization depth of submerged macrophytes was around 5.5 m and about 20 km^2^ of the lake littoral area was covered by submerged macrophytes (Figure 1) and the community was dominated by canopy-forming *P. maackianus* and rosette-type *V. natans* in 2013. *P. maackianus* is a clonal and perennial submerged macrophyte widely distributed in East Asia (Sun, 1992), and forms dense canopies and monocultures in many mesotrophic shallow lakes (Ni, 2001). *V. natans* is a common rosette-type submerged macrophyte species in China. It mainly spreads by clonal reproduction and its aboveground part can overwinter in Lake Erhai.

### Submerged Macrophytes Sampling and Trait Measurements

Submerged macrophyte biomass was collected at 97 transects uniformly distributed perpendicular to the shoreline around Lake Erhai from 16 July to 6 August 2013. Samples were taken at intervals of 1 m water depths along each transect from the shore to the deepest site of macrophyte occurrence (i.e., from around 0.5 m to 5.5 m water depth) (Figure 1). Depending on the macrophyte colonization depth, this resulted in 5 to 6 samples per transect, and in total 527 sampling points. A reaping hook covering a bottom area of 0.2 m^2^ was used to collect submerged macrophytes, with 3 replicates at each sampling point (Figure 1). Plants were washed, sorted by species, and weighed as fresh biomass (FW). GPS coordinates of each sampling point were recorded by a GPS receiver (GPS map 60CSx).

We randomly sampled around 20 full-grown healthy individuals of *P. maackianus* or *V. natans* at each sampling point when the community was dominated by one of these two species and all plant samples were kept in dark containers with ice bags during field work. Three individuals from each sampling point were immediately put into a freezer at −20°C when returning to the laboratory for measuring leaf chlorophyll (chl) *a* content. Part of the mixed freeze-dried leaves from each individual were ground in a mortar and extracted in 96% ethanol for 24 h at 4°C in the dark. The solution was then centrifuged at 4000 × *g* for 10 min, and the chl *a* content determined spectrophotometrically at 665 nm and 649 nm. Plant heights of the remaining individuals were measured and plants were separated into leaves, stems (for *P. maackianus*) or rhizomes (for *V. natans*) and dried at 80°C for 48 h to determine the dry weight (DW).

### Data Analysis

Shoot (leaf+stem/rhizome) chl *a* content is an important indicator for photosynthetic capacity of submerged macrophytes (Nielsen and Sand-Jensen, 1989). We determined leaf chl *a* contents and then related it to the entire shoot biomass by calculating (leaf chl *a* content) × (leaf dry weight)/(leaf dry weight + stem or rhizome dry weight). We used *t*-tests to compare the trait values between the two species and linear regression analysis to evaluate the response of different plant traits to water depth. Plant trait values at each sampling point were represented by the average of all the individuals in that sampling point (i.e., 20 individuals for shoot mass, leaf mass, stem mass, shoot height and 3 individuals for leaf chl *a* and shoot chl *a* content). When plant traits were significantly correlated with water depth for both species, analysis of covariance (ANCOVA) was used to test for differences in the slope of the regression line between the two species. For traits that were correlated with plant size (i.e., leaf biomass, stem biomass, and shoot height), we additionally analyzed biomass-corrected values. To remove differences in size, we conducted a linear regression of each log-transformed trait on log-transformed shoot biomass. The residual values from this regression were saved because they represent size-independent measures of normalized traits (McCoy et al., 2006). We then used linear regression analysis to evaluate the relationship between the residual values and water depth. In this case, the individual trait values were used for the analysis.

GPS coordinates of each sampling point were used to calculate the distance from the shallowest sampling point to the other sampling points in each transect. Then, the littoral slope of each transect was calculated as follows:

(1) D−D1=a×L

(2) Slope=a×100%

Where D is the water depth of the sampling point (except for the shallowest sampling point); D1 is the water depth of the shallowest sampling point; L is the distance from the sampling point to shallowest sampling point in each transect and a is the coefficient of Equation 1. The proportion of the two target species in the total macrophyte biomass (sum of all species) was calculated for each sampling point. In order to compare our results with an earlier study by Duarte and Kalff (1986) on the influence of littoral slopes on biomass of submerged macrophyte communities, we averaged the total macrophyte biomass of all the sampling points in each transect and then examined the relationship between the averaged biomass and the littoral slope. We used the GAM (Generalized Additive Model) to fit the relationship between slope, water depth and total macrophyte biomass as well as proportion of the target species in total macrophyte biomass. GAM is a semi-parametric extension of generalized linear models that enables the user to fit complex non-linear relationships and handle different types of error distributions (Wood, 2006). The models were built with function “gam” in package “mgcv” using penalized regression splines as the smoothing function, Gaussian error distribution, and automatic calculation of smoothing parameters. The main effects and the interaction of the slope and water depth were included in the models. All analyses were performed in R (R Core Team, 2017).

## Results

### Comparison of Plant Traits Between *P. maackianus* and *V. natans* in Lake Erhai

Most of the plant traits measured were significantly different between *P. maackianus* and *V. natans* (Table 1). Shoot biomass of *P. maackianus* (0.54 ± 0.22 g) was lower than that of *V. natans* (0.73 ± 0.36 g), while plant height of *P. macckianus* (187 ± 52 cm) was much higher than that of *V. natans* (115 ± 28 cm). Leaf biomass of *P. maackianus* (0.20 ± 0.14 g) was much lower than that of *V. natans* (0.63 ± 0.2 g). Inversely, the stem biomass of the *P. maackianus* (0.34 ± 0.12 g) was much higher than rhizome biomass of *V. natans* (0.09 g). The leaf chl *a* content of the two species was similar (*V. natans* 7.1 ± 2.0 mg g^−1^, *P. maackianus* 7.2 ± 2.2 mg g^−1^); however, the chl *a* content per shoot dry weight of *P. maackianus* (2.5 ± 0.9 mg g^−1^) was much lower than that of *V. natans* (6.3 ± 2.0 mg g^−1^) (Table 1).

**Table 1:** Comparison between traits values (means ± SD) of *Potamogeton maackianus* and *Vallisneria natans* from Lake Erhai.

Variable	*P. maackianus*		*V. natans*		Significance
	*n*	Mean ± SD	*n*	Mean ± SD	
Shoot biomass (g DW)	278	0.55 ± 0.31	519	0.73 ± 0.36	^∗∗∗^
Leaf biomass (g DW)	278	0.20 ± 0.19	519	0.64 ± 0.33	^∗∗∗^
Stem/rhizome biomass (g DW)	278	0.35 ± 0.17	519	0.09 ± 0.07	^∗∗∗^
Leaf/stem or rhizome biomass ratio	278	0.59 ± 0.40	519	9.47 ± 5.53	^∗∗∗^
Plant height (cm)	278	187.2 ± 57.1	519	114.7 ± 33.8	^∗∗∗^
Leaf chlorophyll *a* content (mg g^−1^ DW)	48	7.21 ± 2.20	87	7.08 ± 1.99	NS
Shoot chlorophyll *a* content (mg g^−1^ DW)	48	2.53 ± 0.90	87	6.26 ± 1.95	^∗∗∗^

*Significance of differences was tested using t-test. DW: dry weight. Significance: ^∗^P < 0.05, ^∗∗^ P < 0.01, ^∗∗∗^ P < 0.001, NS = not significant, n = sample size.*

## Relationship Between Trait Values and Water Depth of *P. maackianus* and *V. natans*

**FIGURE 2:**
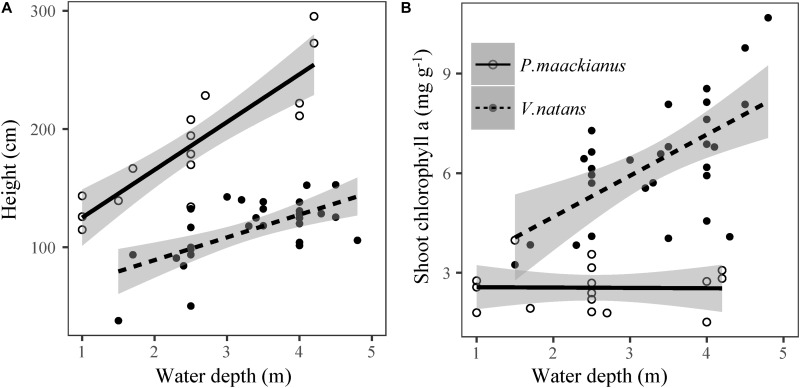
Relationship between plant height **(A)** and shoot chlorophyll *a* content **(B)** of *Potamogeton maackianus* and *Vallisneria natans* and water depth in Lake Erhai. The lines are predicted by linear models.

Plant height of *P. maackianus* were higher than that of *V. natans* at a given water depth (Figure 2). Plant height of both species increased linearly with water depth, but the slope of the regression line of *P. maackianus* was steeper than that of *V. natans* (*t* = −2.94, *p* < 0.01). Leaf chl *a* contents were significantly positively correlated with water depth for both *P. maackianus* and *V. natans* (Table 2). Shoot chl *a* content of *P. maackianus* was stable with increasing water depth, while shoot chl *a* content of *V. natans* increased strongly. *P. maackianus* allocated less biomass into leaves with increasing water depth while the opposite was true for *V. natans* (Table 2).

**Table 2:** Relationship between plant traits of *Potamogeton maackianus* and *Vallisneria natans* and water depth in Lake Erhai based on linear models.

Trait	*P. maackianus*			*V. natans*		
	*n*	*r*^*2*^	Regression coefficient	*n*	*r*^*2*^	Regression coefficient
Shoot biomass (g DW)	16	0.037	−0.035^NS^	29	0.002	−0.012^NS^
Leaf biomass (g DW)	16	0.146	−0.051^NS^	29	0.005	0.015^NS^
Stem biomass (g DW)	16	0.025	0.017^NS^	29	0.240	−0.027^∗∗^
Plant height (cm)	16	0.751	40.391^∗∗∗^	29	0.375	19.206^∗∗∗^
Corrected leaf biomass	278	0.129	−0.143^∗∗∗^	519	0.232	0.045 ^∗∗∗^
Corrected stem/rhizome biomass	278	0.202	0.085^∗∗∗^	519	0.232	−0.290 ^∗∗∗^
Corrected plant height	278	0.700	0.223 ^∗∗∗^	519	0.380	0.213 ^∗∗∗^
Leaf chlorophyll *a* content (mg g^−1^ DW)	16	0.135	1.015^∗^	29	0.319	1.729^∗∗∗^
Shoot chlorophyll *a* content (mg g^−1^ DW)	16	0.000	−0.011^NS^	29	0.347	1.240^∗∗∗^

*Significance: ^∗^P < 0.05, ^∗∗^P < 0.01, ^∗∗∗^P < 0.001, NS = not significant, n = sample size, r^2^ = coefficient of determination, DW = dry weight.*

The corrected stem biomass and plant height of *P. maackianus* increased with water depth and the reverse was found for corrected leaf biomass (Table 2). While corrected rhizome biomass of *V. natans* decreased with water depth, the reverse was found for leaf mass and height (Table 2).

### Distribution and Abundance of *P. maackianus* and *V. natans* in the Littoral of Lake Erhai

**FIGURE 3:**
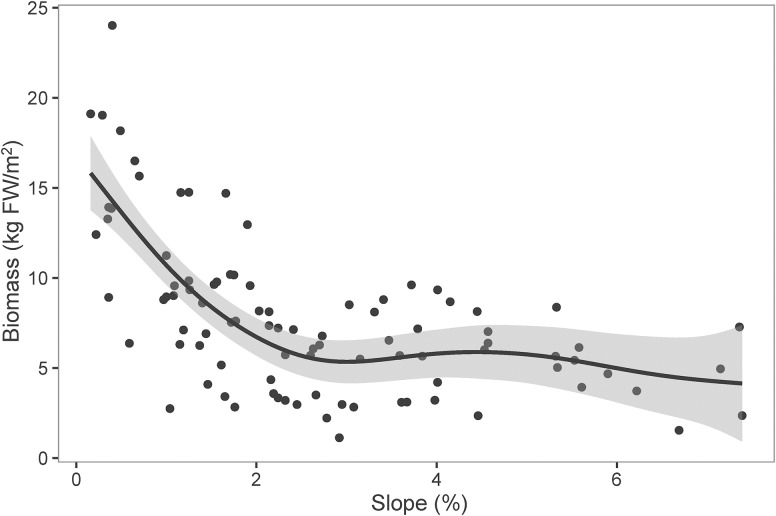
Total biomass of submerged macrophytes (FW: fresh weight) along littoral slopes of Lake Erhai. The line is predicted by Generalized Additive Model (*n* = 97).

**FIGURE 4:**
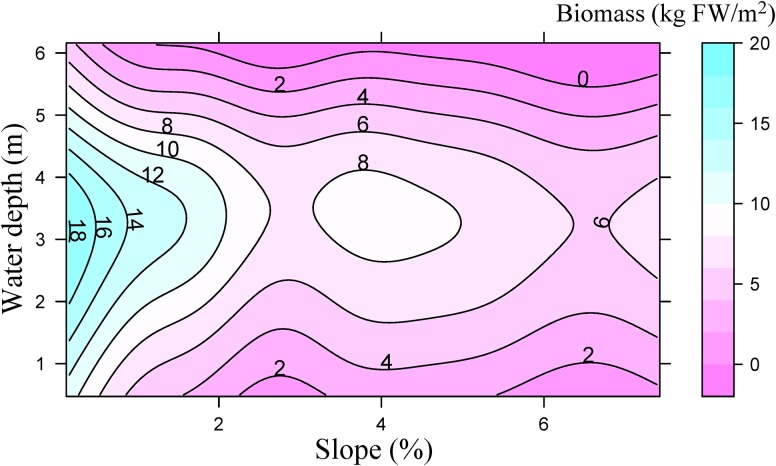
Total biomass of submerged macrophytes (kg fresh weight m^−2^) depending on water depth and littoral slope of Lake Erhai based on a Generalized Additive Model (*n* = 527).

**FIGURE 5:**
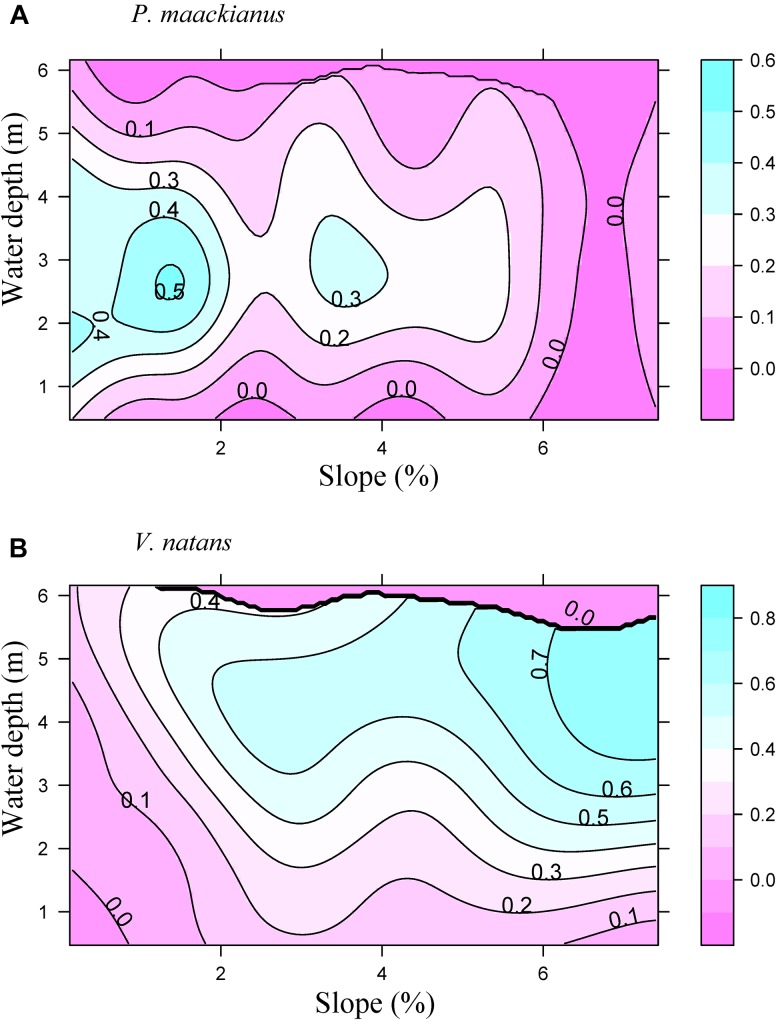
Proportion of the submerged macrophyte species *P. maackianus* **(A)** and *V. natans* **(B)** in the total macrophyte biomass along the water depth and littoral slope of Lake Erhai based on a Generalized Additive Model (*n* = 527).

Littoral slope explained about 53% of the variation of the averaged total macrophyte biomass of transect in Lake Erhai (Table 3). The averaged total macrophyte biomass of transect decreased with basin slope when slopes were lower than 2% (Figure 3), but was relatively stable with increasing slopes above 2%. The combination of littoral slope, water depth and their interaction explained 40% of the variation of the total macrophyte biomass of sampling point (Table 3). The highest biomass of submerged macrophytes appeared at a low slope (0–1%) and moderate water depth (2–4 m) (Figure 4). The GAM model explained 21% and 27% of the variation of the proportion of *P. maackianus* and *V. natans* in the total macrophyte biomass, respectively (Table 3). The proportion of *P. maackianus* in the total macrophyte biomass decreased with increasing littoral slope (Figure 5A), whereas the proportion of *V. natans* increased with increasing littoral slope (Figure 5B).

**Table 3:** Results of GAMs.

Model parameter	gam(ATB)∼s(S)	gam(TB)∼s(S)+s(W)+s(S,W)	gam(PP)∼s(S)+s(W)+s(S,W)	gam(PV)∼s(S)+s(W)+s(S,W)
Deviance explained %	52.9	40.2	20.7	26.8
R^2^ adj.	0.51	0.38	0.17	0.25
N	97	527	527	527
Smooth terms F/edf				
s(S)	19.24/3.96	3.33/6.51	1.05/7.56	2.34/1.00
s(W)	−	5.91/2.67	0.00/1.00	2.78/1.00
s(S,W)	−	0.52/5.76	2.67/13.84	1.60/14.31

*ATB, averaged total macrophyte biomass of transect; TB, total macrophyte biomass of sampling point; PP, proportion of Potamogeton maackianus; PV, proportion of Vallisneria natans; S, slope; W, water depth; edf, estimated degrees of freedom of smoothing function.*

## Discussion

Our data on morphological and physiological traits and distribution of two abundant macrophyte species with different growth forms in Lake Erhai confirmed both initial hypotheses. Both tested species show response strategies to low light conditions typical for canopy-forming (*P. maackianus*) and rosette-type (*V. natans*) macrophyte species (Table 4) and these response strategies significantly influenced their distribution along different water depth and basin slopes.

**Table 4:** Response strategies of canopy-forming and rosette-type submerged macrophytes to low light conditions.

Species	Growth form	Response strategies	Study location	Study type	Reference
*Potamogetonm aackianus*	Canopy-forming	stem elongation; increasing specific leaf area; allocating less biomass to leaves and roots	Lake Erhai, China	Controlled experiment	Fu et al., 2012
*Potamogetonma ackianus*	Canopy-forming	stem elongation; increasing leaf chlorophyll content	Lake Donghu, China	Controlled experiment	Ni, 2001
*Potamogeton maackianus*	Canopy-forming	stem elongation; decreasing branch numbers, minimum saturating irradiance and maximum relative electron transport rate	Lake Donghu, China	Controlled experiment	Chen et al., 2016
*Potamogeto nwrightii*	Canopy-forming	stem elongation; increasing specific leaf area; allocating less biomass to leaves and roots	Lake Erhai, China	Controlled experiment	Fu et al., 2012
*Myriophyllum spicatum*	Canopy-forming	stem elongation; decreasing branch numbers; allocating less biomass to belowground parts	Lake Krankesjon, Sweden	Controlled experiment	Strand and Weisner, 2001
*Vallisneria natans*	Rosette-type	increasing shoot height; decreasing minimum saturating irradiance and maximum relative electron transport rate	Lake Donghu, China	Controlled experiment	Chen et al., 2016
*Vallisneria natans*	Rosette-type	increasing shoot height; allocating more biomass to leaves; allocating less biomass to roots	Lake Erhai, China	Controlled experiment	Fu et al., 2012
*Vallisneria americana*	Rosette-type	increasing shoot height, leaf chlorophyll content and photosynthetic efficiency	Gloucester Point, Virginia	Controlled experiment	French and Moore, 2003

## Response Strategies of Submerged Macrophytes to Low Light Conditions

Stem elongation was found as a major response strategy to low light conditions by the canopy-forming species *P. maackianus*, while the rosette-type species *V. natans* produced more chl *a* per shoot biomass at deeper sites to tolerate light attenuation. These findings are consistent with a similar study by Chen et al. (2016) assessing the response of *P. maackianus* and *V. natans* to low light conditions in Lake Donghu, China. In our study, *V. natans* increased leaf chl *a* content and allocated more biomass to leaves at deep sampling points, resulting in a strong increase of chl *a* content per shoot biomass. Such strategy combined with a low light compensation point for photosynthesis (Chen et al., 2016), allows *V. natans* to successfully survive at sites with low light conditions in Lake Erhai and colonize deep areas with high slopes. Other rosette-type species such as *V. americana* have shown a similar morphological and physiological response to low light conditions (French and Moore, 2003). This indicates that rosette-type species may mainly adopt a tolerance strategy towards stress in low light conditions (Table 4). In contrast, *P. maackianus* responds to low light conditions mainly through allocating more shoot biomass into stems to concentrate leaves closer to the water surface. In our study, leaf chl *a* content of *P. maackianus* slightly increased with water depth, but the chl *a* content per shoot biomass did not increase with water depth as less leaf biomass formed at deeper sites. This shade avoidance strategy has also been found in terrestrial plants (Franklin, 2008) and seems common among canopy-forming submerged species (Table 4).

### Response to Low Light Conditions Affects Macrophyte Distribution Along Basin Slopes and Water Depth

The alternative response strategies to low light conditions of *P. maackianus* and *V. natans* affected their ability to grow at different basin slopes and water depths. The canopy-forming species *P. maackianus* was dominant at sites with low-moderate water depth (2–4 m) and low-moderate basin slopes (0–4%). In contrast, the rosette-type *V. natans* prevailed at deeper sites (3–5 m) with higher slopes (4%–7%). The potential mechanism allowing *V. natans* growing at deeper waters than *P. maackianus* is that photosynthetic adjustments would become more important in determining plant abundance in deep water due to the lower carbon requirements compared with shoot elongation (Chen et al., 2016).

Previous studies suggested the plant communities may be governed by a dominance–tolerance trade-off, where most species perform best in benign, productive sites (i.e., undisturbed sites with a high availability of resources); however, there is often a trade-off between the ability to dominate at productive sites or to sequester high-quality resources and the ability to persist on low-quality resources or to tolerate harsh conditions (Wisheu and Keddy, 1992; McGill et al., 2006). Lake eutrophication enhances the availability of nutrients both in sediment and water column which initially increases the competition for light between the submerged macrophytes. As a result, short-growing species (e.g., charophytes and rosette-type angiosperms) are commonly replaced by canopy-forming species (Hilt et al., 2018), which can form dense monocultures in mesotrophic lakes (He et al., 2015). However, high slope areas in mesotrophic lakes may provide a refuge for short-growing species due to a trade-off between the ability to compete for light and the ability to tolerate harsh conditions like strong drag forces and nutrient-poor sediment at high slope areas. As a consequence of the different response strategies to low light conditions, *P. maackianus* plants are taller than *V. natans* in a given water depth and therefore have an advantage in competing for light. However, this strategy and their thin stems result in a lower resistance of *P. maackianus* to drag forces by currents and waves (Puijalon et al., 2011; Fu et al., 2014). In contrast, *V. natans* has ribbon-like leaves growing close to the bottom, which are more resistant to drag than *P. maackianus* in a given flow velocity (Puijalon et al., 2011). The nutrient and organic matter content in sediment in low slope areas are not assumed to limit the growth of *V. natans* since this species has been shown to grow on a wide range of sediment types in this lake (He et al., 2017). For instance, the total nitrogen, total phosphorus and organic matter contents in sediments were around 3, 0.7, and 100 mg g^−1^, respectively in Haichao bay of Lake Erhai (unpublished data), all of which are suitable for growth of *V. natans* (Xiao et al., 2007). However, the macrophyte community was dominated by *P. maackianus* rather than *V. natans* in this area, which indicated the *V. natans* might be excluded by competition other than sediment nutrient content in low slope areas. Consequently, this trade-off between the response strategy to low light conditions and hydrodynamic disturbance resistance likely determines the distribution and abundance of the two major macrophyte species with different growth forms in Lake Erhai. Still, other mechanisms such as wind or other sediment characteristics may contribute to the observed distribution of the two species (Schutten et al., 2004, 2005).

Our finding of a significantly decreased total macrophyte biomass with increasing basin slope is consistent with previous observations made in temperate lakes by Duarte and Kalff (1986). Total macrophyte biomass in Lake Erhai decreased significantly at littoral slopes above 2%. Our data suggest that the different response of macrophyte species to low light conditions may contributes to this pattern. In the 1970s and 1980s, submerged macrophytes covered around 40% of Lake Erhai, and the dominant species were *Hydrilla verticillata* and *V. natans.* Macrophyte coverage decreased from 40% to 8% from 1980s to 2012 due to eutrophication. During this period, most of the short species such as charophytes and *V. natans* were lost in deeper areas and *H. verticillata* was replaced by *P. maackianus* (Fu et al., 2013). However, *V. natans* still occupies large areas of the littoral zone in this lake, especially in high slope areas, confirming our finding that these habitat conditions may provide a niche for survival of this species. Similar displacements of rosette species have been found in temperate lakes, where *Isoetes* tend to grow deep under oligotrophic conditions, but are similarly displaced as *V. natans* to the littoral zone with strong disturbance during eutrophication (Arts, 2002).

We conclude that the different response strategies of submerged macrophytes with different growth forms (rosette-type versus canopy-forming) to low light conditions might significantly affect their distribution and abundance in lakes along gradients of other stressors such as physical forces. Morphological heterogeneity of lakes may thus contribute to the maintenance of a high diversity of submerged macrophytes, especially under mesotrophic conditions where competition for light between macrophytes is particularly relevant (Salgado et al., 2017).

## Author Contributions

TC and LN conceived the idea and proposed the method. LH, TZ, YW, WL, HZ, and XZ contributed to conduct the sampling and traits measurements. LH, TC, XZ, and SH wrote the manuscript. All authors read and approved the final manuscript.

## Conflict of Interest Statement

The authors declare that the research was conducted in the absence of any commercial or financial relationships that could be construed as a potential conflict of interest. The handling Editor is currently organizing a Research Topic with one of the authors TC and confirms the absence of any other collaboration.
